# Association between ultra-processed food intake and risk of colorectal cancer: a systematic review and meta-analysis

**DOI:** 10.3389/fnut.2023.1170992

**Published:** 2023-07-06

**Authors:** Long Shu, Yiqian Huang, Caijuan Si, Qin Zhu, Peifen Zheng, Xiaoyan Zhang

**Affiliations:** ^1^Department of Nutrition, Zhejiang Hospital, Hangzhou, Zhejiang, China; ^2^Department of Anesthesia Surgery, Zhejiang Hospital, Hangzhou, Zhejiang, China; ^3^Department of Digestion, Zhejiang Hospital, Hangzhou, Zhejiang, China

**Keywords:** ultra-processed food, NOVA, colorectal cancer, meta-analysis, systematic review

## Abstract

**Background:**

Although some epidemiological studies have shown a positive relationship between high intake of ultra-processed food (UPF) and risk of colorectal cancer (CRC), the results remain inconsistent. Therefore, we conducted this systematic review and meta-analysis to clarify the association between UPF intake and CRC risk.

**Methods:**

PubMed/MEDLINE, Web of Science, EMBASE, China National Knowledge Infrastructure (CNKI) and Wan fang databases were used to search the relevant studies published up to February 2023. The summary relative risks (RRs) with the corresponding 95% confidence intervals (CIs) were estimated by comparing the highest category vs. the lowest category of UPF intake, using the random-effects models (DerSimonian-Laird method). Heterogeneity between studies was explored using the Cochran’s Q test and I-square (I^2^). Publication bias was assessed by examining the funnel plots, and quantified by Begg’s or Egger’s tests.

**Results:**

A total of seven articles (three cohort and four case-control studies), involving 18,673 CRC cases and 462,292 participants, were included in our study. Combining nine effect sizes from seven articles, an increased risk of CRC was shown in the highest compared with the lowest category of UPF intake (RR = 1.26; 95%CI:1.14–1.38, *p* < 0.0001). Subgroup analyses showed a positive association between UPF intake and CRC risk in case–control studies (RR = 1.41; 95%CI: 1.22–1.63, *p* < 0.0001). When we conducted analyses separately by study area, there was a significant association between UPF intake and CRC risk in developed countries (RR = 1.20; 95%CI: 1.11–1.30, *p* < 0.0001).

**Conclusion:**

Our results show that high UPF intake is significantly associated with a higher risk of CRC, in the absence, however, of a dose–response association. Further studies in particular of large prospective cohort studies are necessary to confirm these results.

## Introduction

Colorectal cancer (CRC) is the third most commonly diagnosed cancer globally and the second leading cause of cancer-related deaths in 2020, with more than 1.9 million new cases and 93,500 deaths (1). Although the incidence rates of CRC have decreased in some high-risk countries due to effective cancer screening measures, it still accounts for about 10% of annually cancer cases and cancer-related deaths (2). In China, parallel with rapid economic development and changes in eating habits, the incidence and mortality rates of CRC have been steadily rising (1, 3). According to the latest International Agency for Research on Cancer (IARC) estimates, CRC remains the second most frequent cancer in China, after lung cancer, and is responsible for approximately 0.56 million new cases and 0.29 million deaths in 2020 (1). Meanwhile, CRC is also the third most commonly diagnosed malignancy in the United States (4), with an estimated 1.5 million new cases and 53,200 cancer deaths in 2020 (5). As far as we know, genetic and environmental risk factors play the key role in the progression of CRC (2, 6). Among environmental risk factors, diet has been acknowledged as an important modifiable risk factor for the primary prevention of CRC (7).

During the past few decades, some epidemiological studies have examined the relations between diets intake and risk of CRC (8–10), but these associations have mostly been explored through dietary quality indices (10), individual nutrients/foods (8), or dietary pattern analyze (9). There was compelling evidence from the World Cancer Research Fund/American Institute for Cancer Research (WCRF/AICR) continuous update project on the relationship between foods and beverages and CRC risk also concluded that processed meats and alcoholic drinks could increase the risk of CRC (11). Additionally, based on the reports from the American Cancer Society (ACS), higher consumption of red and processed meats, and lower consumption of dietary fiber have been recognized as one of risk factors for CRC (12). Together, research associating individual nutrients or food groups and risk of CRC has been widely explored (8–11), but the association between degree of food processing and CRC risk has been poorly studied. Since 2009, the NOVA food classification system was developed by Brazilian researchers to enable categorization of food item according to the degree of processing (13). In this NOVA food classification system, foods and food products are divided into four groups, including unprocessed or minimally processed food, processed culinary ingredients, processed food and ultra-processed foods (UPFs) (14). Such UPF is energy-dense food, usually high in added sugar, salt, saturated and trans fats, and low in dietary fiber, vitamins and minerals (15). Currently, the global consumption of UPF has been rising in most middle- and high-income countries, contributing to 25%~60% of total energy intake (16–18). In the United Kingdom, UPF consumption has already represented 65.4% of energetic intake in children, 67.8% in adolescents and 54.3% in adults (17). These trends have coincided with a shift in many countries toward diets associated with a rising prevalence of chronic diseases (18), triggering the growing interest in researchers to explore the associations between consumption of UPF and various health outcomes.

In the last decade, with the advent of global interest on UPF, there has been growing evidence that UPF intake is associated with poor health outcomes, including increased risks of obesity, hypertension, diabetes, cardiovascular disease and all-cause mortality (19–24). At the same time, several recent systematic review and meta-analyses have also been published to evaluate the relationship between UPF intake and risks of overweight/obesity, type 2 diabetes, hypertension and all-cause mortality (15, 25–27). Unsurprisingly, high UPF intake has been shown to be positively related to these adverse health outcomes. To date, however, only few epidemiological studies have specially explored the relationship between consumption of UPF and CRC risk (18, 28–33), yielding the inconsistent results. Some observational studies have shown a positive relationship between high consumption of UPF and CRC risk (29, 30, 32, 33), whereas other studies reported a null finding (18). Furthermore, to our knowledge, no previous meta-analysis to date has comprehensively assessed the association between consumption of UPF and risk of CRC. Therefore, to determine the impact of UPF intake on CRC risk, we carried out this comprehensive systematic review and meta-analysis to synthesize these findings of observational studies published from inception to February 2023.

## Materials and methods

### Search strategy

The current systematic review and meta-analysis was performed according to the Preferred Reporting Items for Systematic Reviews and Meta-Analyses guidelines (34). A systematic literature search was originally conducted in December 2022, and was updated in February 2023, using five electronic databases including PubMed/MEDLINE, Web of Science, EMBASE, CNKI and Wan fang, without limitations on language and publication date. The following keywords or phrases were utilized in the search strategies: (“fast food” OR “processed food” OR “ultra-processed food” OR “processed meat” OR “hamburger” OR “salami” OR “bacon” OR “sausage” OR “luncheon meats”) AND (“colorectal cancer” OR “colorectal neoplasia” OR “colorectal adenomas” OR “rectum cancer” OR “rectal cancer” OR “colon cancer”). Moreover, the reference lists from the retrieved articles and systematic reviews were checked for additional relevant studies. Unpublished studies or gray literature were not included in this meta-analysis. The literature search was conducted by two independent authors (Shu L and Huang YQ). Disagreements were resolved by consensus after discussion with another author (Zhang XY).

### Study selection

Two authors (Shu L and Huang YQ) independently screened and crosschecked each article from the literature search, and a third author (Zhang XY) was consulted to settle any discrepancies. After screening the titles and abstracts, the full-text versions of the articles were reviewed according to the inclusion and exclusion criteria of the present meta-analysis. Studies were included in our analyses if they met all the following eligibility criteria: (1) were observational studies (cross-sectional, case–control or cohort studies); (2) were performed in humans of any age; (3) UPF was recognized as the main exposure (according to the NOVA food classification system); (4) evaluated the association with CRC risk; (5) reported adjusted estimates of the RRs [e.g., hazard ratios (HRs) or odds ratios (ORs)] and 95%CIs for the relationship between UPF intake and CRC risk; (6) If the original published data lacked sufficient detail, the corresponding author of the study is contacted by email for more information. Studies were excluded if they met one of the following criteria: (1) non-observational studies, e.g., reviews, editorials, case reports and conference letters; (2) animal, cell culture, and *in vitro* studies; (3) did not use the NOVA food classification system (assessed the only specific food or food groups, such as processed meat); (4) studies not reported as HRs, RRs or ORs with 95%CIs; (5) unrelated articles.

### Data extraction

Data were extracted by two independent authors from all eligible studies, including first author’s last name, publication year, study design, study area, sample size, number of CRC cases, mean age, duration of follow-up, method of UPF assessment, and confounding factors used for adjustments in the multivariable analyses.

### Definition and determining the intake of ultra-processed food

According to the extent and purpose of industrial food processing, the NOVA food classification system divided foods and food products into four groups: unprocessed or minimally processed food, processed culinary ingredients, processed food and UPFs (14). UPFs are industrial formulations entirely or mostly from substances derived from additives (e.g., flavorings, colorings, emulsifiers,) and foods, containing little or no whole food (15). These food products are typically ready-to-eat, hyper-palatable, and characterized by high energy density, added sugar, salt, saturated and trans fats, and low amounts of dietary fiber, vitamins and minerals (15). Examples of UPF include ice cream, cookies, soft drinks, cakes, pizza, instant noodles, hamburger, and smoking meats.

### Quality assessment

The authors (Huang YQ and Si CJ) independently evaluated each included study’s quality using the Newcastle-Ottawa Scale (NOS), which was designed for case-control and cohort studies (35). In the NOS checklist, scores ranged from 0 to 9 based on the eight items related to study selection (4 stars), comparability of participants (2 stars), and assessment of outcome/exposure of interest (3 stars). Finally, those studies with NOS scores ≥7 points were deemed as high quality (36). Any discrepancies between two authors were resolved by a third author (Shu L) to reach a consensus.

### Data synthesis and statistical analyses

In this study, data were measured as log RR with standard errors (SEs) by using the ORs, HRs, RRs and their corresponding 95%CIs. The pooled effect sizes and 95%CIs were estimated comparing the highest vs. the lowest category of UPF intake, using the random-effects models. Heterogeneity among the included studies was examined by the Cochran’s Q test and I-squared (I^2^) statistics. *p*-values of Cochran’s Q test < 0.10 or I^2^ > 50% were considered to show significant heterogeneity among the included studies, and subsequently the random-effects models (DerSimonnian and Laird method) were used to summary the pooled RRs. Otherwise, the fixed-effects models were performed (37). According to the I^2^ value, heterogeneity was classified as low (I^2^ ≤ 25%), moderate (25%~75%) and high (I^2^ ≥ 75%), respectively. If the results showed significant heterogeneity (I^2^ > 50%), the potential sources of heterogeneity across studies were explored using subgroup and sensitivity analyses. In our analyses, subgroup analyses were performed based on study design (cohort or case–control studies), outcomes (colorectal cancer or colorectal adenomas), study quality (≥7 or <7), mean age (≥55 or <55), study area (developed countries or developing countries), sample size (<5,000 or >5,000), and exposure assessment (FFQ or 24 h dietary recall). Sensitivity analysis was performed, excluding one study removed at one time to confirm whether the results were robust or sensitive to the influence of single study. Publication bias was assessed by examining the funnel plots, and quantified by Begg’s or Egger’s tests (38). If publication bias was observed, the effect size was re-estimated using the trim and fill method (39). All statistical analyses were conducted using STATA version 14.0 (StataCorp, College Station, TX, United States), with a 2-sided *p*-value < 0.05 showing statistical significance.

## Results

### Overview of included studies for this systematic review

**Figure 1:**
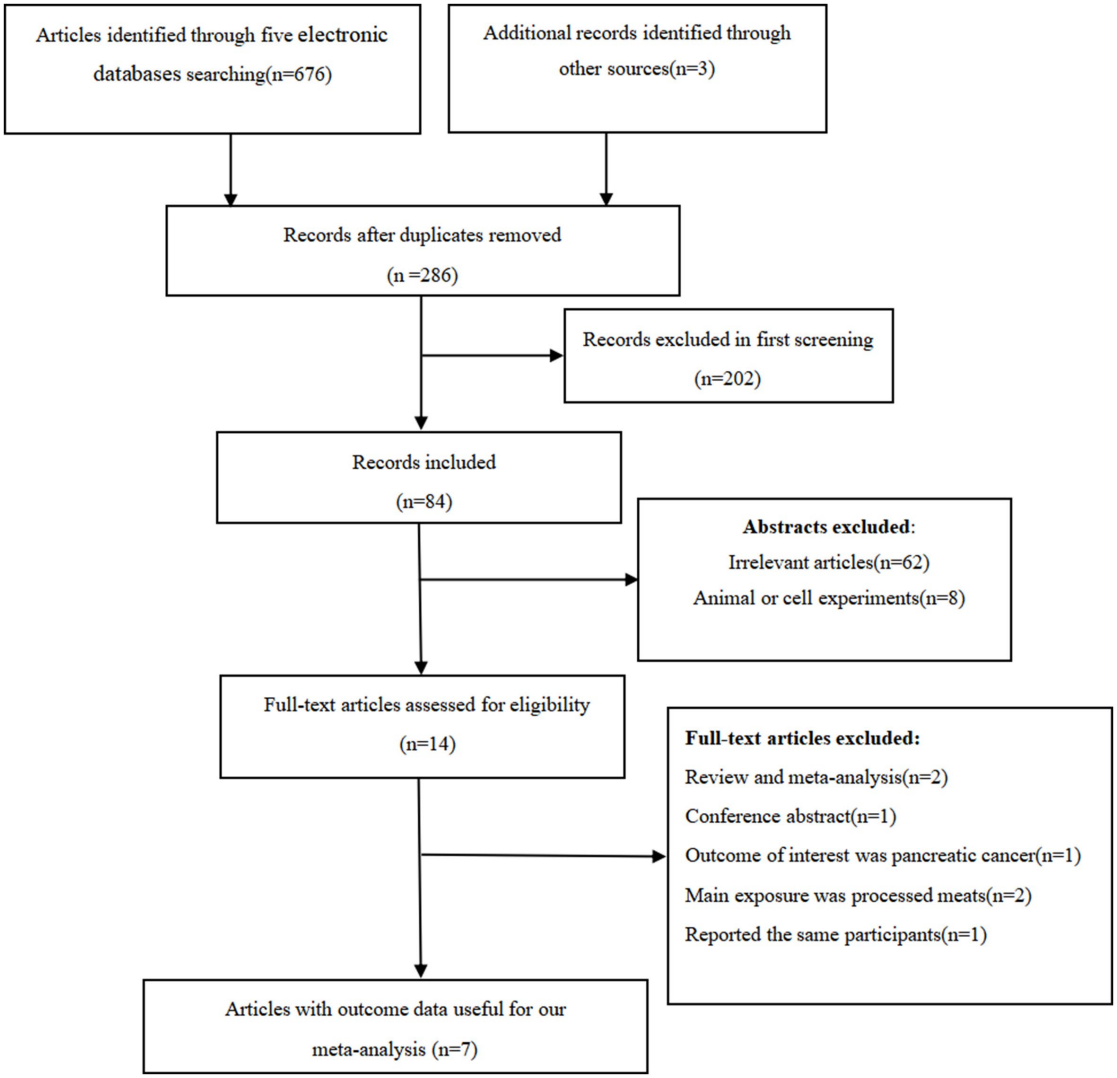
Flow chart of article screening and selection process.

Figure 1 indicates the flowchart of the selection of the articles. We identified 679 articles through database search and reference lists of relevant articles. After the removal of duplicates, 286 articles remained for further screening. Subsequently, 202 articles were excluded basing on first screening. After reviewing the titles and abstracts, 14 full-text articles were independently reviewed in details by two authors and subsequently seven articles were excluded because of the following reasons: systematic review or meta-analyses (*n* = 2), the outcome of interest was pancreatic cancer (*n* = 1), the main exposure was processed meats (*n* = 2), conference abstract (*n* = 1), and reported the same participants (*n* = 1). Finally, seven articles with 9 effect sizes were included in the present meta-analysis.

### Characteristics of the studies

The main characteristics of included studies are summarized in Table 1. In total, seven articles with 462,292 participants and 18,673 CRC cases were included in this meta-analysis. Out of seven included studies, four of them were case-control studies (28, 29, 31, 32), and three were cohort studies (18, 30, 33). All included studies were published in English between 2018 and 2022. Two of the included studies were performed in United States (30, 33), one in Spain (28), one in Israel (29), one in France (18), one in Morocco (31), and one in Iran (32). All participants were 18 years and older. The follow-up duration for cohort studies ranged from 18 to 28 years. Sample size ranged from 213 to 159,907. All included studies classified the UPF according to the NOVA food classification system, and reported in both sexes (18, 28–33). Data on dietary intake was collected using 24-h dietary recalls (18) or food frequency questionnaires (28–33). Overall, based on the NOS scores, five of all the included studies were classified as of high quality (18, 28, 30, 32, 33), and the remaining two were of medium-quality (29, 31).

**Table 1:** Characteristics of included studies on the association between ultra-processed food intake and risk of colorectal cancer.

References	Location	Study design	Total number of participants	Age	Exposure assessment	Adjustment or matched for in analyses	RR (95%CI) for highest vs. lowest category
Wang et al. (33)	United States	Cohort	206,248	25–75 years	FFQ	Age, calendar year of current questionnaire, race, family history of cancer, history of endoscopy, total alcohol intake, physical activity, smoking status and pack years of smoking, total caloric intake, and regular aspirin use and additionally for menopausal status and postmenopausal hormone use in women.	Male:1.29(1.08–1.53)
			(3,216 cases)				Female:1.04(0.90–1.20)
Romaguera et al. (28)	Spain	Case-control	1,842 cases	20–85 years	FFQ	Sex, age, study area, educational level, body mass index, physical activity, smoking, nonsteroidal anti-inflammatory drugs, family history of colorectal cancer, total energy intake, and ethanol intake.	Male:1.34(1.10–1.65)
			5,241 controls				Female:1.24(0.96–1.59)
Fliss-Isakov et al. (29)	Israel	Case-control	294 cases	40–70 years	FFQ	Age, gender, BMI, total kcal, aspirin use and indication for colonoscopy.	1.75 (1.14–2.68)
			358 controls				
Fiolet et al. (18)	France	Cohort	104,980 (153 cases)	≥18 years	24-h dietary recall	Age, sex, energy intake without alcohol, number of 24 h dietary records, smoking status, educational level, physical activity, height, body mass index, alcohol intake, family history of cancers, intakes of lipids, sodium, and carbohydrates and Western dietary pattern (derived by factor analysis).	1.16(0.95–1.42)
EI Kinany et al. (31)	Morocco	Case-control	1,453 cases	≥18 years	FFQ	Age, education level, family history of CRC, smoking status, physical activity, BMI and total energy intake.	1.40(1.22–1.61)
			1,453 controls				
Jafari et al. (32)	Iran	Case-control	71 cases	45–65 years	FFQ	Matched the patient on age (within 5 years) and race (black or white).	3.32(1.44–7.61)
			142 controls				
Hang et al. (30)	United States	Cohort	142,052 (11,644 cases)	25–75 years	FFQ	Age, race, cohort, time period of endoscopy, number of prior endoscopies, time in years since the most recent endoscopy, family history of colorectal cancer, total alcohol intake, physical activity, smoking status and pack-years of smoking, regular aspirin use, additionally for menopausal status, and postmenopausal hormone use (never or ever) in women.	1.18(1.11–1.26)

BMI, Body mass index; CRC, Colorectal cancer; FFQ, Food frequency questionnaire; USA, United states.

### Ultra-processed food intake and CRC risk

**Figure 2:**
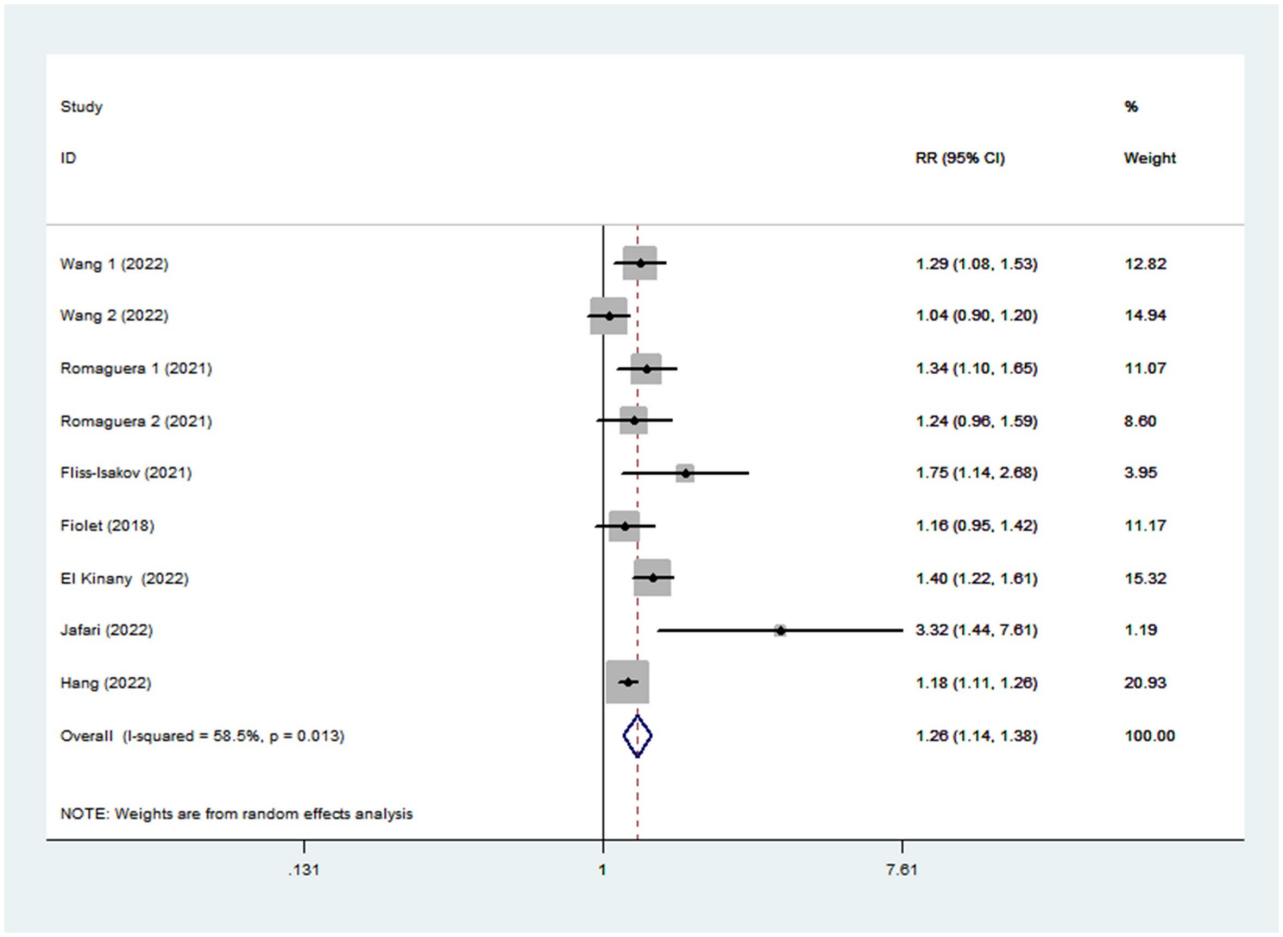
Forest plot of the association between consumption of UPF and CRC.

Seven articles reporting nine original studies were included in this meta-analysis. Figure 2 showed a significantly increased risk of CRC in the highest compared with the lowest categories of UPF intake (RR = 1.26; 95%CI: 1.14–1.38; *p* < 0.0001). The moderate heterogeneity across studies was found (I^2^ = 58.5%, *p* = 0.013), and data from these studies was evaluated using the random-effects models.

### Subgroup analyses

**Figure 3:**
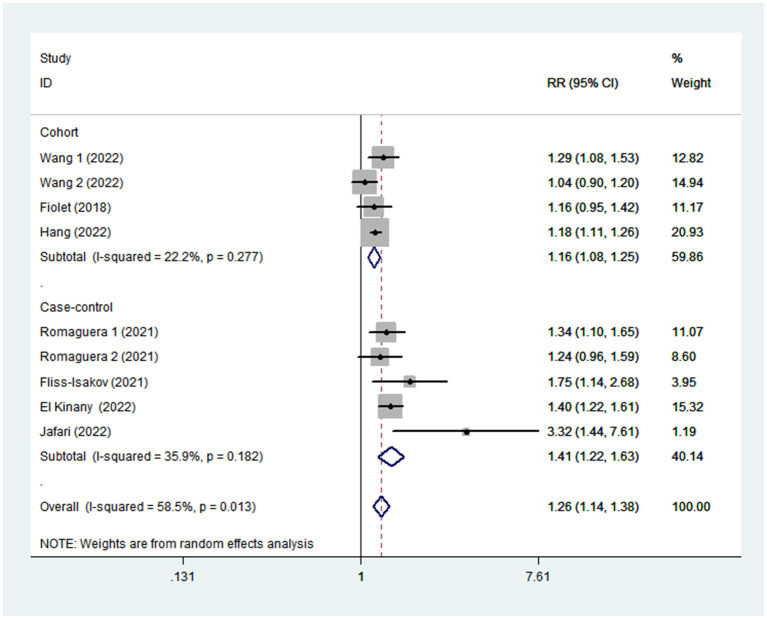
Forest plot of the association between consumption of UPF and CRC risk stratified by study design.

**Figure 4:**
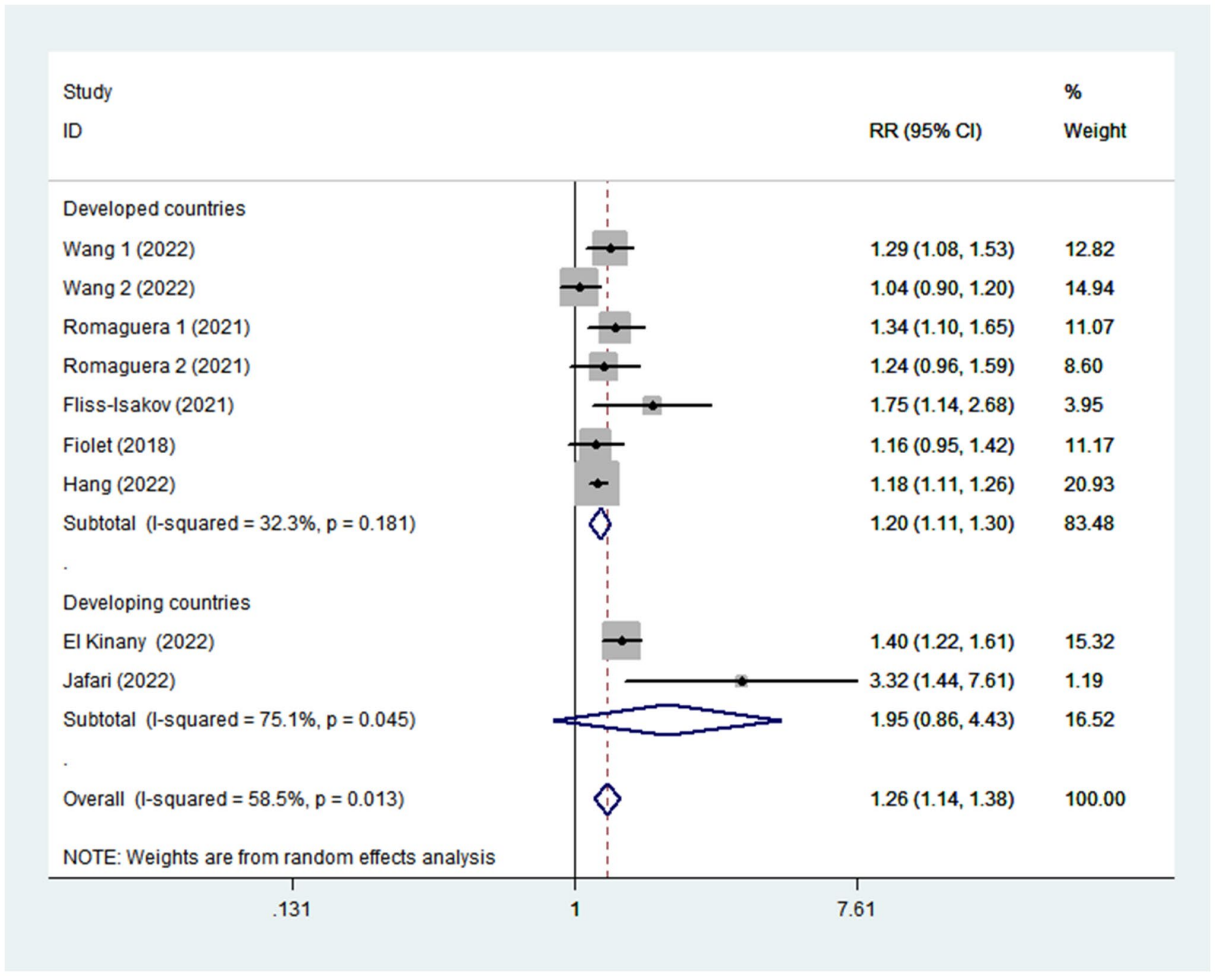
Forest plot of the association between consumption of UPF and CRC risk stratified by study area.

**Figure 5:**
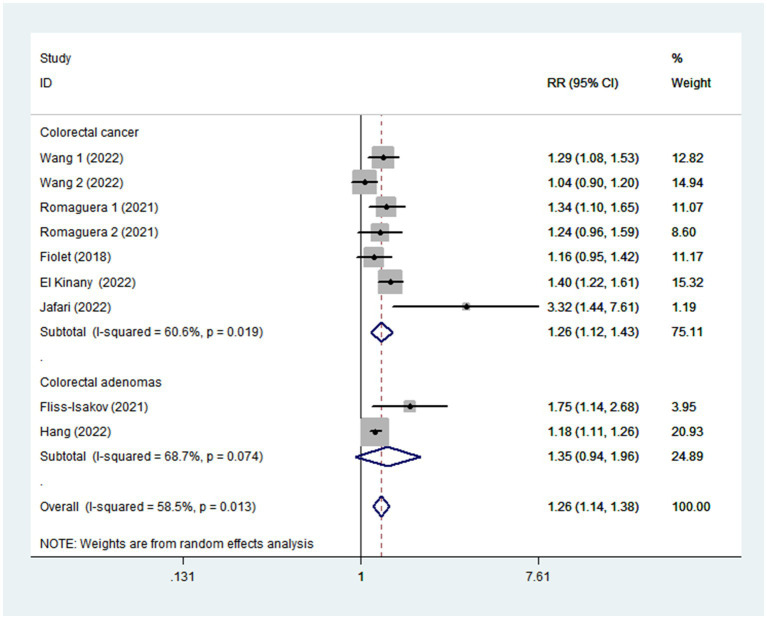
Forest plot of the association between consumption of UPF and CRC risk stratified by study outcome.

**Figure 6:**
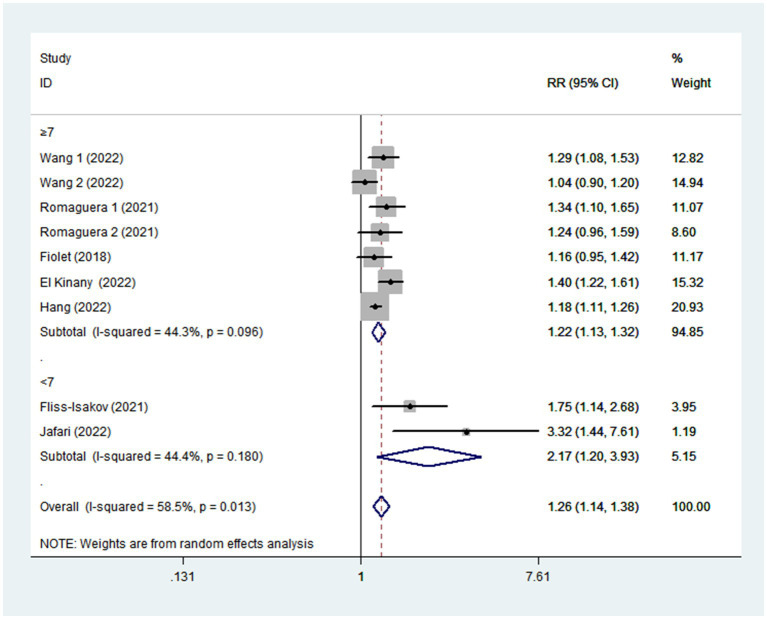
Forest plot of the association between consumption of UPF and CRC risk stratified by study quality.

**Figure 7:**
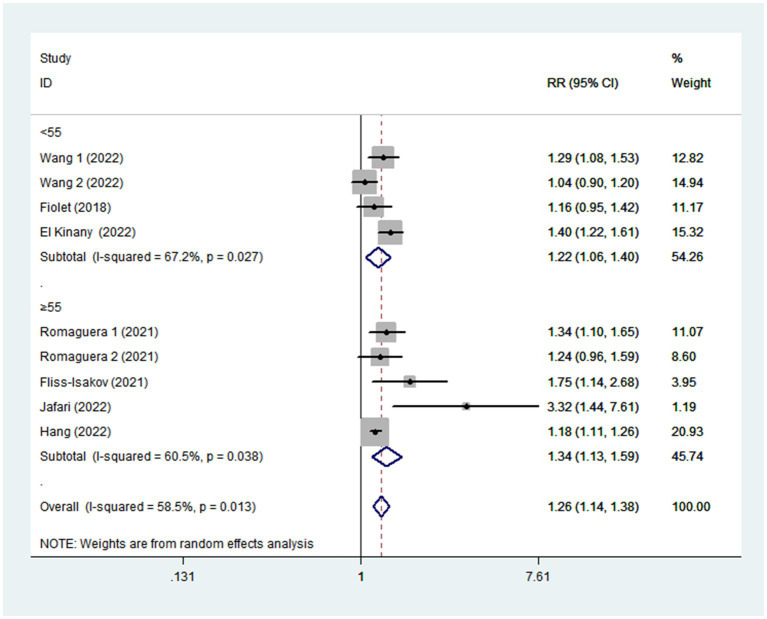
Forest plot of the association between consumption of UPF and CRC risk stratified by mean age.

**Figure 8:**
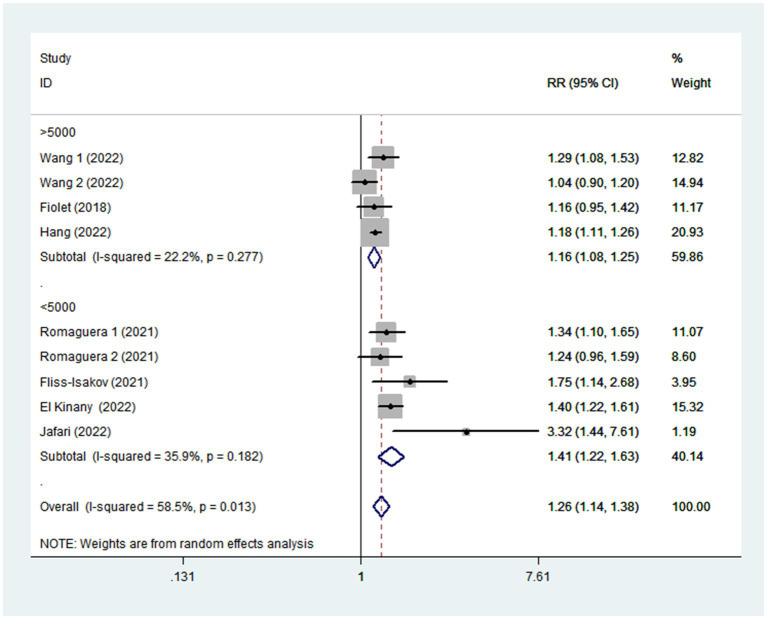
Forest plot of the association between consumption of UPF and CRC risk stratified by sample size.

**Figure 9:**
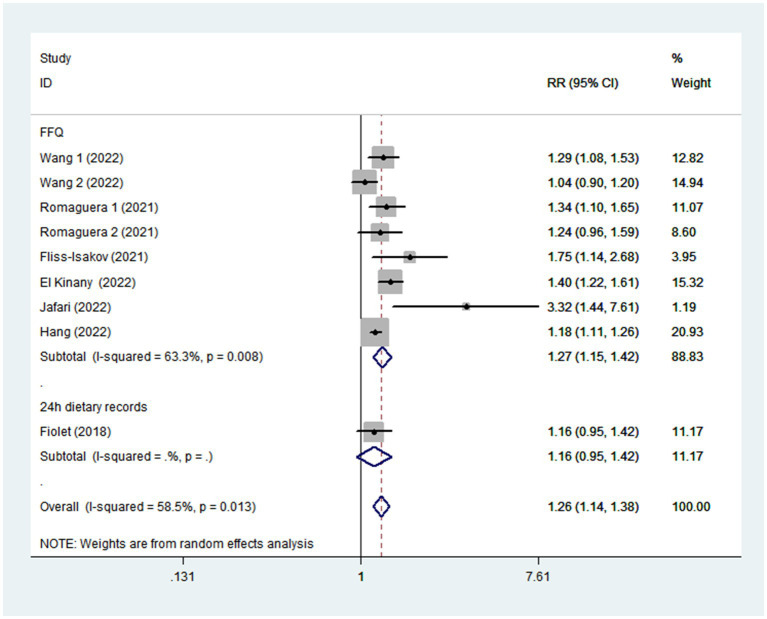
Forest plot of the association between consumption of UPF and CRC risk stratified by exposure assessment.

Given the moderate heterogeneity of this meta-analysis (I^2^ = 58.5%; *p* = 0.013), subgroup analyses were carried out to explain the potential sources of heterogeneity (Table 2). In our study, subgroup analyses were stratified basing on the study design (cohort/case-control studies), outcomes (colorectal cancer/colorectal adenomas), study quality (≥7/<7), mean age (≥55/<55), study area (developed/developing countries), sample size (<5,000/>5,000), and exposure assessment (FFQ/24 h dietary recall). When we analyzed study design separately (Figure 3), results showed a positive relationship between UPF intake and CRC risk in cohort studies (RR = 1.16; 95%CI: 1.08–1.25, *p* < 0.0001). There was no evidence of heterogeneity between studies (*p =* 0.277; I^2^ = 22.2%). Meanwhile, there was a more significant relationship between UPF intake and CRC risk in case–control studies (RR = 1.41; 95%CI: 1.22–1.63, *p* < 0.0001), with less evidence of heterogeneity (*p =* 0.182; I^2^ = 35.9%). For study area (Figure 4), there was a significant association between UPF intake and risk of CRC (RR = 1.20; 95%CI: 1.11–1.30, *p* < 0.0001) in developed countries and the between-studies heterogeneity decreased from 58.5% to 32.3%. However, no statistical association was observed between UPF intake and risk of CRC in developing countries (RR = 1.95; 95%CI: 0.86–4.43, *p* = 0.112). For study outcome (Figure 5), we found that a positive association between UPF intake and risk of CRC in the subgroups of colorectal cancer (RR = 1.26; 95%CI:1.12–1.43, *p* < 0.0001). However, the heterogeneity was apparent (*p* = 0.019, I^2^ = 60.6%). In contrast, we found no significant association between UPF intake and CRC risk in the subgroups of colorectal adenomas (RR = 1.35; 95%CI: 0.94–1.96, *p* = 0.106), and there was more heterogeneity (*p =* 0.074; I^2^ = 68.7%). For study quality (Figure 6), UPF intake was statistically significant in the subgroups of study quality ≥ 7 (RR = 1.22; 95%CI: 1.13–1.32, *p* < 0.0001) and <7 (RR = 2.17; 95%CI: 1.20–3.93, *p* = 0.010). However, heterogeneity between studies decreased 58.5 to 44.3%. When the results were stratified by mean age (Figure 7), a positive association between UPF intake and risk of CRC was observed in the subgroups of age < 55 (RR = 1.22; 95%CI:1.12–1.40, *p* = 0.007), and there was evidence of significant heterogeneity (*p =* 0.027; I^2^ = 67.2%). Moreover, we also observed the significant positive association between UPF intake and CRC risk in the subgroups of age ≥ 55 (RR = 1.34; 95%CI:1.13–1.59, *p* = 0.001). The stratified association between UPF intake and CRC risk according to sample size based on the random-effects model is shown in Figure 8. There was less evidence of heterogeneity in sample size > 5,000 (*p =* 0.277; I^2^ = 22.2%), where significant positive association with risk of CRC was observed (RR = 1.16; 95%CI:1.08–1.25, *p* < 0.0001). In addition, significant positive association was also found between UPF consumption and CRC risk in the studies with sample size < 5,000 (RR = 1.41; 95%CI: 1.22–1.63, *p* < 0.0001), and there was less heterogeneity (*p* = 0.182, I^2^ = 35.9%). For exposure assessment (Figure 9), there was a marginally significant association between UPF intake and risk of CRC (RR = 1.27; 95%CI: 1.15–1.42, *p* < 0.0001) in FFQ, and the heterogeneity was more heterogeneity (*p =* 0.008; I^2^ = 63.3%).

**Table 2:** Subgroup analyses for the association between ultra-processed food intake and risk of colorectal cancer.

Study characteristic	Category	No. of studies	RR (95%CI)	*p*
Study design	Case-control	4	1.41(1.22–1.63)	<0.0001
	Cohort	3	1.16(1.08–1.25)	<0.0001
Exposure assessment	FFQ	6	1.27(1.15–1.42)	<0.0001
	24 h dietary recall	1	1.16(0.95–1.42)	0.148
Outcomes	Colorectal cancer	5	1.26(1.12–1.43)	<0.0001
	Colorectal adenomas	2	1.35(0.94–1.96)	0.106
Study quality	≥7	5	1.22(1.13–1.32)	<0.0001
	<7	2	2.17(1.20–3.93)	0.010
Mean age	≥55	4	1.34(1.13–1.59)	0.001
	<55	3	1.22(1.12–1.40)	0.007
Study area	Developed countries	5	1.20(1.11–1.30)	<0.0001
	Developing countries	2	1.95(0.86–4.43)	0.112
Sample size	<5,000	4	1.41(1.22–1.63)	<0.0001
	>5,000	3	1.16(1.08–1.25)	<0.0001

FFQ, Food frequency questionnaire; RR, relative risk; CI, confidence interval.

### Publication bias and sensitivity analysis

As shown in Supplementary Figure 1, examination of the funnel plots revealed little evidence of asymmetry. Begg’s and Egger’s tests for publication bias had no statistical significance (highest vs. lowest categories of UPF consumption: Begg’s test: *p* = 0.118; Egger’s test: *p* = 0.083).

### Sensitivity analysis

Based on the results of sensitivity analysis (Supplementary Figure 2), the relationship between high intake of UPF and CRC risk was not affected by any single study or a couple of studies.

### Quality assessment

The quality of the included studies using NOS standards is presented in Table 3. When included studies received a NOS score of seven or higher, they would be considered of high quality (18, 28, 30, 32, 33). Besides, the remaining two articles were identified as medium-quality (29, 31).

**Table 3:** Ultra-processed food intake and risk of colorectal cancer: assessment of Study Quality.

Studies	Selection					Comparability			Outcome			Score
	1	2	3	4		5A	5B		6	7	8	
**Cohort**												
Wang et al. (33)	*	*	*	*		*	*		*	*	*	9
Fiolet et al. (18)	*	*	*	*		*			*	*	*	8
Hang et al. (30)	*	*	*	*		*	*		*	*	*	9
**Case-control**												
Romaguera et al. (28)	*	*	*			*	*		*	*		7
Fliss-Isakov et al. (29)	*	*				*			*	*		5
El Kinany et al. (30)	*	*	*	*		*			*	*		7
Jafari et al. (31)	*	*	*			*			*	*		6

* For case-control studies, 1 indicates cases independently validated; 2, cases are representative of population; 3, community controls; 4, controls have no history of blood pressure disease; 5A, study controls for age; 5B, study controls for additional factor(s); 6, ascertainment of exposure by blinded interview or record; 7, same method of ascertainment used for cases and controls; and 8, non-response rate the same for cases and controls. For cohort studies, 1 indicates exposed cohort truly representative; 2, non-exposed cohort drawn from the same community; 3, ascertainment of exposure; 4, outcome of interest not present at start; 5A, cohorts comparable on basis of age; 5B, cohorts comparable on other factor(s); 6, quality of outcome assessment; 7, follow-up long enough for outcomes to occur; and 8, complete accounting for cohorts.

## Discussion

To our knowledge, this systematic review and meta-analysis is the first to comprehensively evaluate the relationship between UPF intake and CRC risk. In this study, results showed a positive relationship between high intake of UPF and CRC risk. Nevertheless, the included studies must be interpreted with caution due to moderate heterogeneity in the results of this meta-analysis. To address the heterogeneity, we conducted subgroup analyses based on study design, study area, exposure assessment, study outcomes, study quality and sample size. In addition, sensitivity analysis also showed that none of included studies could considerably modify the summary effect. Our findings confirm the positive correlation results of previous studies and add to the growing evidence for the impact of high UPF intake on diet-related CRC, despite the lack of a dose–response relationship.

In China, with an estimate 0.56 million new cases and 286,162 deaths, CRC is reported to be the second most commonly diagnosed cancer and the fifth leading cause of cancer death (40). Considering the high incidence of CRC and its increasing burden on public health, there is an urgent need to identify the possible causes of this disease. Increasing evidence from observational studies suggest that dietary factors are the important modifiable risk factors for CRC (33). However, in the past few decades, diets in many countries have changed considerably, typified by substituting UPFs for fresh or minimally processed foods. In this study, we found a significant positive relationship between UPF intake and CRC risk, although there was evidence of moderate heterogeneity across studies (I^2^ = 58.5%; *p* = 0.013). Our results are in agreement with some previous studies reporting that high consumption of UPF was associated with an increase in the risk of CRC (29, 30, 32, 33). In three large United States prospective cohorts, Wang and colleagues found that high consumption of total UPF in men was associated with an increased risk of CRC (RR = 1.29, 95%CI: 1.08–1.53), and certain subgroups of UPF (ready-to-eat/heat mixed dishes) in women was associated with an elevated risk of CRC (RR = 1.17, 95%CI: 1.01–1.36) over the follow-up period of 24–28 years (33). Similarly, a recent case-control study conducted in Israel medical center, Fliss-Isakov et al., also reported that high intake of UPF was strongly associated with colorectal adenomas (OR = 1.75; 95%CI:0.14–2.68) (29). Conversely, in the French NutriNet-Santé cohort, Fiolet and colleagues failed to find a significant association between UPF intake and risk of CRC (RR = 1.16, 95%CI: 0.95–1.42) (18). These discrepant results across studies may be due to the differences in assessment method of UPF consumption, the amounts and types of UPF consumed within different populations, and duration of study follow-up. On the one hand, the amounts and types of UPF consumption across different countries could be different. For example, it is reported that approximately 60% of total energy intake was consumed in United States (33), and 21.5% of daily energy intake was consumed in Brazil (14). On the other hand, most studies used FFQs to collect the data on UPF consumption (28–33) and only one study used 24-h dietary recalls (18). Moreover, a longer duration of study follow-up may be required for the harmful effect of UPF intake to become apparent. Although evidence on the relationship of UPF consumption with risk of CRC remains inconclusive, several potential mechanisms may explain this observed association. First, high consumption of UPF has been associated with higher risks of weight gain and obesity, all of which are well-known risk factors of CRC (41). Second, UPF often contains high content of added sugars and saturated fat. As reported in a previous study, high consumption of added sugar could cause changes in intestinal microbiota (42), which has been identified as a key factor in the development of CRC (43). Third, food processing and preparation, particularly high-temperature treatment may produce some neoformed contaminants found in UPF, such as acrylamide, which has been classified by the IARC as a Group 2A carcinogen (probably carcinogenic to humans) (44). Also of note, acrylamide has already been clarified as carcinogenic and genotoxic by the European Food Safety Agency in 2015 (45). Fourth, potential carcinogens may be formed during meat processing, such as sodium nitrite. Accumulating evidence has suggested that sodium nitrite, the preservative and coloring substance in processed meats (an important part of UPF), may increase the risk of CRC (46). Likewise, in the 2015 IARC report, processed meat consumption has also been classified as a Group 1 carcinogen (47). Fifth, beyond nutritional aspects, UPF usually contains some food additives that may be involved in progression of CRC. For example, dietary emulsifiers (e.g., polysorbate-80 and carboxymethylcellulose) and artificial sweeteners (e.g., saccharin) could alter the gut microbiome, thereby promoting inflammation and colonic carcinogenesis (48, 49). In addition, titanium dioxide (TiO_2_), a common food additive, is used as a brightening agent or in contact with food or drinks packaging to provide better texture and antimicrobial properties. TiO_2_ is currently assessed by the WHO and IARC as “probably carcinogenic to humans” in relation to cancer (group 2B) (50). Sixth, UPF’s plastic packaging may contain some carcinogenic substances that come into contact with food, such as bisphenol A, which has been judged as “a substance of very high concern” by the European Chemicals Agency (51). A recent experimental study in cellular models showed that bisphenol A plays an important role in the development and progression of colon cancer (52). Finally, the adverse effect of UPF intake on CRC may partly be attributed to lower intake of vegetables, fruits, legumes, and whole grains, which are well-known sources of dietary fiber. A recent systematic review and meta-analysis has provided the convincing level of evidence for an inverse relationship between dietary fiber intake and risk of CRC (53). Together, above-mentioned these mechanisms may explain why UPF intake has been linked to an increased risk of CRC.

In our analyses, the results showed the moderate between-study heterogeneity on the relationship between UPF intake and CRC risk (I^2^ = 58.5%, *p* = 0.013). Hence, we conducted subgroup analyses based on study design (cohort or case-control studies), exposure assessment (FFQ or 24 h dietary recall), outcomes (colorectal cancer or colorectal adenomas), study quality (≥7 or <7), mean age (≥55 or <55), study area (developed countries or developing countries), and sample size (<5,000 or >5,000) to explore the sources of heterogeneity. Our results showed that difference in study design, study area and sample size might partially explain the heterogeneity, whereas mean age and study quality had no significant effect. For example, the heterogeneity of cohort studies decreased from 58.5% to 22.2%. There are several possible explanations for the observed heterogeneity. First, 4 of 7 studies were case-control in term of study design. In case-control studies, recall bias due to dietary survey methods (i.e., FFQ and 24-h dietary recall) should be considered. Moreover, there were only three prospective cohort studies included, which somewhat limited the significance of the pooled results. Second, although the RRs or ORs were from the highest vs. lowest categories of UPF intake, different studies classified UPF intake based on different standards, such as absolute intake (18), percentage (%) of daily total energy (kcal) (30) or servings per day (33). These may cause substantial heterogeneity. Third, the different models included in the study to control for potential confounding variables, may explain the heterogeneity observed in our analyses. In the included studies, adjustment for potential confounding variables were inconsistent. Consequently, we inevitably have high level of heterogeneity when combining studies. Fourth, the moderate heterogeneity would exist because the follow-up time was different, which might affected the outcome. Prospective cohort studies with long-term follow-up might establish temporality between UPF intake and risk of CRC. Ultimately, there was still considerable heterogeneity in the subgroup analyses, suggesting the presence of other unknown confounding factors.

### Strengths and limitations

This study has its own advantages. First, this is the first comprehensive systematic review and meta-analysis, to our knowledge, that assessed the association between UPF intake and CRC risk. Our findings add to the growing body of evidence for the role of UPF intake in diet-related CRC and help inform public policy for CRC prevention. Second, articles were strictly selected according to pre-determined inclusion criteria, including only studies whose UPF classification faithfully followed the characteristics proposed by the NOVA system. Third, CRC cases were ascertained through medical records and pathological reports by clinicians, reducing the risk of misclassification. Fourth, the quality of included studies was medium to high, and the pooled RRs were multivariate and adjusted for some known confounders. Meanwhile, we also performed subgroup and sensitivity analyses to explore the potential sources of heterogeneity. Fifth, there was no significant evidence of publication bias in the funnel plot, and the statistical test (e.g., Begg’s and Egger’s tests) for publication bias was non-significant. Despite the above-mentioned strengths, several limitations also should be acknowledged in the present study. First, in our meta-analysis, more than half of included studies were case–control in design. We cannot rule out the probability that these findings are susceptible to recall and selection bias. Second, six of the included studies used FFQs that were not specially designed or validated to evaluate UPF consumption, thus misclassification might occur when authors classified food items into the given food groups. Also, misclassification might result in the under- or over-estimation of UPF intake. Third, even though some known risk factors have been adjusted in the analyses, residual confounding from unmeasured factors cannot be totally excluded. Moreover, there were also different adjustment for potential confounding factors in the included studies. Consequently, the data included in our meta-analysis might have varying degrees of completeness and accuracy. Fourth, moderate heterogeneity was found in this meta-analysis. Although subgroup and sensitivity analyses were performed to explore the potential sources of heterogeneity, we could not ascertain and explain the sources of inter-study heterogeneity sufficiently. Finally, the majority of studies in this meta-analysis were performed in developed countries (e.g., France and United States), with only two studies in developing countries, which might compromise the generalizability of our findings.

## Conclusion

In conclusion, this study indicate that high intake of UPF is associated with a higher risk of CRC. Despite the lack of a dose-response relationship between UPF intake and CRC risk, our findings still suggest that high intake of UPF may be an important public health issue in the prevention and management of CRC, and support the need to emphasize the importance of limiting UPF intake for better health outcomes in national dietary guidelines. Moreover, our findings may also help physicians and dietitians in clinical practice by provide strong evidence on the role of UPF intake in the management of CRC. Future research priorities included well-designed prospective studies exploring the relationship between UPF intake and CRC risk in different populations around the world.

## Data availability statement

The original contributions presented in the study are included in the article/Supplementary material, further inquiries can be directed to the corresponding author.

## Author contributions

XZ designed the research. LS performed the systematic literature search, identified the studies meeting the inclusion criteria, extracted data from the included studies, and drafted the manuscript. CS acquired the data. YH evaluated the risk of bias of the included studies. QZ performed the statistical analysis. XZ and PZ assisted in the interpretation of the results and the revision of the manuscript. All authors contributed to the article and approved the submitted version.

## Funding

This work was supported by the National Natural Science Foundation of China (grant number: 82004040), Traditional Chinese Medicine Research Project of Zhejiang (no. 2020ZB009, 2021ZB010), and Medical and Health research fund project of Zhejiang Province (no. 2022KY006).

## Conflict of interest

The authors declare that the research was conducted in the absence of any commercial or financial relationships that could be construed as a potential conflict of interest.

## Publisher’s note

All claims expressed in this article are solely those of the authors and do not necessarily represent those of their affiliated organizations, or those of the publisher, the editors and the reviewers. Any product that may be evaluated in this article, or claim that may be made by its manufacturer, is not guaranteed or endorsed by the publisher.

## Abbreviations

ACS: American Cancer Society
AICR: American Institute for Cancer Research
CI: Confidence interval
CNKI: China National Knowledge Infrastructure
CRC: Colorectal cancer
FFQ: Food frequency questionnaire
HR: Hazard ratio
IARC: International Agency for Research on Cancer
NOS: Newcastle-Ottawa Quality Scale
OR: Odds ratio
RR: Relative risk
SEs: Standard errors
UPF: Ultra-processed food
WCRF: World Cancer Research Fund
