# Supplementary figures and images for: Association between ultra-processed food intake and risk of colorectal cancer: a systematic review and meta-analysis
Source: Front Nutr. 2023 Jul 6;10:1170992. doi: 10.3389/fnut.2023.1170992 (PMC10358360; doi:10.3389/fnut.2023.1170992)

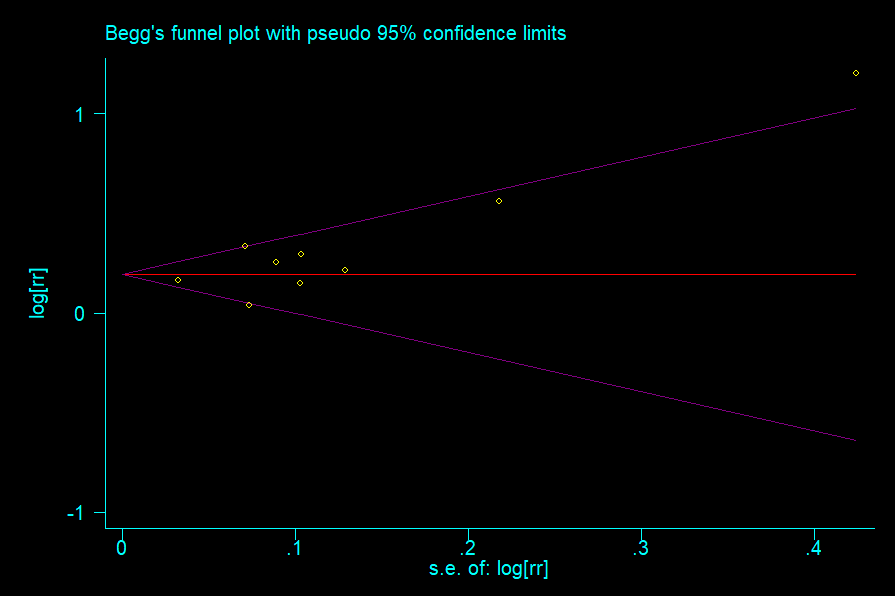

Supplement: Supplementary file 1 [file Image_1.TIF]

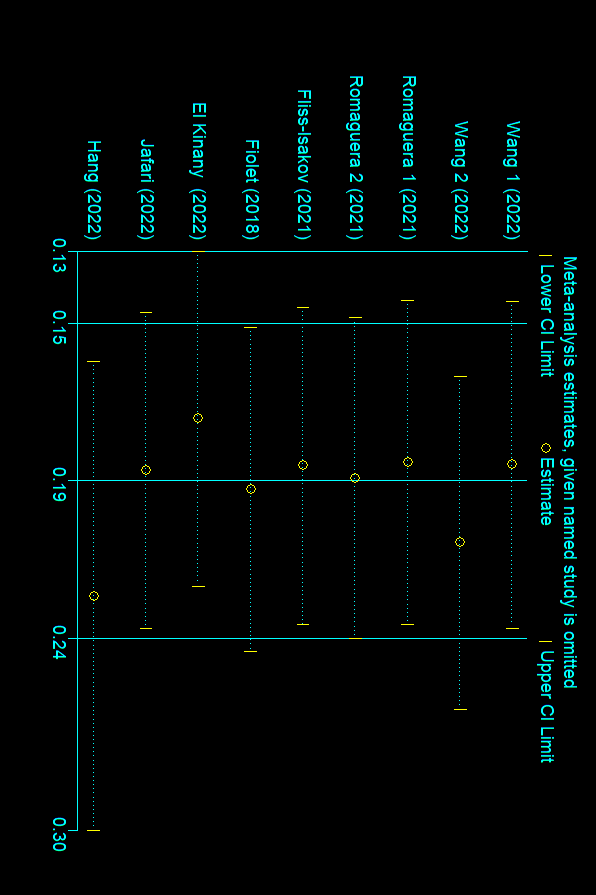

Supplement: Supplementary file 2 [file Image_2.TIF]
